# Optical coherence tomography reflects clinically relevant gray matter damage in patients with multiple sclerosis

**DOI:** 10.1007/s00415-022-11535-8

**Published:** 2023-01-10

**Authors:** Alessandro Cagol, Nuria Cerdá Fuertes, Marc Stoessel, Muhamed Barakovic, Sabine Schaedelin, Marcus D’Souza, Jens Würfel, Alexander U. Brandt, Ludwig Kappos, Till Sprenger, Yvonne Naegelin, Jens Kuhle, Cristina Granziera, Athina Papadopoulou

**Affiliations:** 1 Translational Imaging in Neurology (ThINK) Basel, Department of Biomedical Engineering, Faculty of Medicine, University Hospital Basel and University of Basel, Basel, Switzerland; 2 Department of Neurology, University Hospital Basel, Petersgraben 4, 4031 Basel, Switzerland; 3 Research Center for Clinical Neuroimmunology and Neuroscience Basel (RC2NB), University Hospital Basel and University of Basel, Basel, Switzerland; 4 Department of Clinical Research, University Hospital and University of Basel, Basel, Switzerland; 5 Medical Image Analysis Center and Department of Biomedical Engineering, University Basel, Basel, Switzerland; 6 Experimental and Clinical Research Center Max Delbrueck Center for Molecular Medicine, Charité Universitätsmedizin Berlin, Berlin, Germany; 7 University of Irvine, Irvine, CA USA; 8 Department of Neurology, DKD Helios Klinik Wiesbaden, Wiesbaden, Germany

**Keywords:** MS, OCT, Ganglion cells, MRI, Neurodegeneration, Brain atrophy

## Abstract

**Background:**

Retinal degeneration leading to optical coherence tomography (OCT) changes is frequent in patients with multiple sclerosis (PwMS).

**Objective:**

To investigate associations among OCT changes, MRI measurements of global and regional brain volume loss, and physical and cognitive impairment in PwMS.

**Methods:**

95 PwMS and 52 healthy controls underwent OCT and MRI examinations. Mean peripapillary retinal nerve fiber layer (pRNFL) thickness and ganglion cell/inner plexiform layer (GCIPL) volume were measured. In PwMS disability was quantified with the Expanded Disability Status Scale (EDSS) and Symbol Digit Modalities Test (SDMT). Associations between OCT, MRI, and clinical measures were investigated with multivariable regression models.

**Results:**

In PwMS, pRNFL and GCIPL were associated with the volume of whole brain (*p* < 0.04), total gray matter (*p* < 0.002), thalamus (*p* ≤ 0.04), and cerebral cortex (*p* ≤ 0.003) –both globally and regionally–, but not white matter. pRNFL and GCIPL were also inversely associated with T2-lesion volume (T2LV), especially in the optic radiations (*p* < 0.0001). The brain volumes associated with EDSS and SDMT significantly overlapped with those correlating with pRNFL and GCIPL.

**Conclusions:**

In PwMS, pRNFL and GCIPL reflect the integrity of clinically-relevant gray matter structures, underling the value of OCT measures as markers of neurodegeneration and disability in multiple sclerosis.

**Supplementary Information:**

The online version contains supplementary material available at 10.1007/s00415-022-11535-8.

## Introduction

Neuroaxonal loss plays a crucial role in multiple sclerosis (MS) pathophysiology showing close association with clinical disability [1]. However, direct in vivo monitoring of neuroaxonal loss remains challenging.

Optical coherence tomography (OCT) offers the opportunity to visualize and quantify layers of neurons and axons in the retina, in a non-invasive and patient-friendly way, with high inter-rater and intra-rater reproducibility [2]⁠. OCT alterations are frequently detected in patients with MS (PwMS) as a consequence of clinically-evident or sub-clinical optic nerve demyelination, retrograde degeneration triggered by tissue damage in the posterior visual pathway, and primary degeneration of ganglion cells in parallel with neuronal degeneration in the rest of the CNS [3]⁠.

Among OCT measures, peripapillary retinal nerve fiber layer (pRNFL) and ganglion cell/inner plexiform layer (GCIPL) are most sensitive and robust in reflecting MS-associated neurodegeneration; conversely, retinal inner nuclear layer (INL) has been associated with inflammatory activity [4].

Previous studies showed consistent associations of OCT measures with white matter lesion load and total brain volume (TBV) loss [5–8]⁠. However, investigations on the relationship of OCT measures with the volumes of gray matter (GM) and white matter (WM), as well as with volumes of regional brain structures relevant in MS pathology, produced contradictory results [6, 8–11]⁠. Conflicting results were also obtained when considering the cerebral cortex regionally, with reported associations either limited to areas involved in the visuospatial performance or concomitantly involving areas relevant for cognitive performance—including the insula [12–14]⁠.

Understanding to what extent OCT measures reflect brain volume loss, not only in the afferent visual pathway, but also in regions critically involved in physical and cognitive performance, can strengthen the use of OCT as a marker of CNS neurodegeneration in MS.

Thus, our aims were:

1. To investigate the association of OCT measures (pRNFL, GCIPL, and INL) with global/regional brain volumes; additionally, to explore the relative strength of association of OCT measures with (i) global neurodegeneration and (ii) WM lesions in the optic radiations (ORs) – as a proxy of retrograde degeneration in the visual pathway;
2. To assess whether OCT measures reflect changes in brain volumetric measurements related to physical and cognitive disability.

## Materials and methods

### Study design

We conducted a cross-sectional study in PwMS and healthy controls (HCs). Demographic and clinical data were collected. PwMS underwent neurological and cognitive screening assessments; both PwMS and HCs underwent OCT and brain MRI examinations. All evaluations were obtained for each participant within two weeks.

The study is reported following the Strengthening the Reporting of Observational Studies in Epidemiology (STROBE) guideline [15]⁠. The study was approved by the local ethics committee (Ethikkommission Nordwest- und Zentralschweiz; 285/11) and conducted in accordance with the declaration of Helsinki. Written informed consent was obtained from all subjects before study enrollment.

### Participants

PwMS were prospectively recruited at the MS Center of the University Hospital of Basel, between 2016 and 2019. A thorough investigation of the past clinical history was performed by neurologists specialized in MS. For inclusion, PwMS had to fulfill the following criteria: 1) diagnosis of MS according to the 2010 revised McDonald criteria; [16]⁠ 2) age ≥ 18 years. The exclusion criteria were: 1) known ophthalmological comorbidities (e.g. glaucoma); 2) refractive errors > 6 diopters; 3) history of bilateral optic neuritis.

HCs were recruited between 2017 and 2019, matched in age and sex with the PwMS. To be enrolled, HCs had to have: 1) no medical history of neurological, psychiatric, and ophthalmological diseases; 2) age ≥ 18 years. The only exclusion criterion was the presence of refractive errors > 6 diopters.

### Clinical assessment

In PwMS, neurological examinations were performed by certified raters to determine the Expanded Disability Status Scale (EDSS) score [17].Cognitive performance was explored with the Symbol Digit Modalities Test (SDMT) [18].⁠ Raw SDMT scores were converted to age, and education-corrected z-scores based on published normative data⁠ [18].

### OCT acquisition and analysis

OCT was performed on a Heidelberg Engineering Spectralis device, in a dark room, without pupil dilation. OCT quality control was performed according to the OSCAR-IB criteria; [19]⁠ all images passed quality assessment. pRNFL was measured using 3.4 mm ring scans around the optic nerve head (12°, 1536 A-scans, 12 ≤ ART ≤ 100); the mean thickness was used in the analysis. Macular data were obtained with a macular volume scan, with eye-tracking (scan details: 25° × 30°, 61 vertical B-scans, 768 A-scans per B-scan). After an initial automated segmentation of all intraretinal layers using the software provided by Heidelberg Engineering (Eye Explorer 1.9.13.0), segmentation results of the macular ganglion cell layer (GCL), the inner plexiform layer (IPL), and the INL were checked, and corrected where needed. For the analyses we used the combined GCIPL volume of the macular region (6-mm-diameter cylinder of the 1, 3, 6 mm ring adjacent to the fovea), as well as the INL volume. 8 subjects had to be excluded for the GCIPL/INL analysis (reasons for exclusion are reported in Online Resource 1).

To selectively investigate the effect of chronic neurodegeneration on OCT measures, excluding changes related to acute focal damage, eyes with history of optic neuritis were excluded (*n* = 31).

### MRI acquisition and analysis

Brain MRI was acquired on a 3-Tesla scanner (Siemens Skyra). The protocol included a 3-dimensional (3D), T1-weighted, 1-mm isotropic magnetization prepared rapid gradient-echo (MPRAGE), and a 3D, 1-mm isotropic fluid-attenuated inversion recovery (FLAIR). All images were visually assessed to ensure absence of image artifacts; 5 scans had to be excluded due to insufficient quality (motion artifacts).

T2-hyperintense lesions were automatically segmented, and results were manually revised. Volumetric analysis was performed on MPRAGE images, after filling WM hypointensities, with *FreeSurfer (version 6.0.0, *http://surfer.nmr.mgh.harvard.edu*)*. Cortical reconstructions and volumetric segmentations were visually inspected, and manually corrected if needed.

As measures of interest we considered total brain volume (TBV), total GM volume, total WM volume, and cerebellar volume, as well as volumes of specific GM structures (namely cerebral cortex, thalamus, caudate, putamen, and hippocampus). Cortical volumes were further investigated regionally, according to the Desikan–Killiany atlas [20]. Total intracranial volume (TIV) was quantified with *SPM12 (*www.fil.ion.ucl.ac.uk/spm/software/spm12*).*

T2-lesion volume (T2LV) in each brain lobe and in the ORs was obtained after co-registering the lesion masks with *FreeSurfer* reconstructions and with the Juelich brain segmentation atlas, [21]⁠ respectively.

### Statistical analysis

Statistical analysis was conducted in *R (version 3.6.3; *http://www.R-project.org*)*.

Comparisons of demographic, clinical, OCT, and MRI measurements between PwMS and HCs were performed with Welch’s t-test, Pearson’s chi-squared test, and Mann–Whitney U test. Due to non-normal distribution, T2LV and EDSS score were logarithmic transformed (T2LV-log; EDSS-log).

Associations between OCT measures and (i) global/regional T2LV-log, (ii) EDSS-log, and (iii) SDMT z-score were explored with univariable linear regression models. Associations between global/regional brain volumes and (i) OCT measures, (ii) EDSS-log, and (iii) SDMT z-score were investigated with general linear models, adjusting for age, sex, total T2LV-log, and TIV.

To assess the relative strength of association of (i) retrograde degeneration along the visual pathway and (ii) global neurodegeneration, with the OCT measures of neuroaxonal loss (RNFL, GCIPL), we performed multivariable regression models with OCT measures as dependent variables, and T2LV-log in ORs (taken as a marker of retrograde degeneration) as well as GM fraction (i.e., the ratio between GM volume and TIV, taken as a marker of global neurodegeneration) as explanatory variables, adjusting for age, and sex.

Results were corrected for multiple comparisons using the false discovery rate (FDR) approach; reported p-values are adjusted for FDR. Effect size is reported as the standardized regression coefficient (β). Graphical results for regional cortical volume analysis were displayed with the *fsbrain* package [22]⁠.

To exclude that the observed OCT-MRI associations were affected by the effect of disease-modifying therapies (DMTs) which are known to have an impact on both MRI and OCT measures [23]⁠—we performed a sensitivity analysis including DMTs class as a covariate in the statistical models.

Additional sensitivity analyses were conducted to explore the OCT-MRI associations (i) separately in patients with relapsing–remitting MS (RRMS) and progressive MS, as well as (ii) in multivariable regression models including also disease duration and EDSS score as covariates (Online Resources 3–6).

## Results

In total, 95 PwMS and 52 HCs were included in the study. Demographic and clinical characteristics of the cohort are summarized in Table 1.

**Table 1:** Main demographic and clinical cohort’s characteristics

	PwMS (*n* = 95)	HCs (*n* = 52)	Between-group comparison
Age (years): mean (SD)	49.9 (11.3)	51.5 (13.6)	*t = 0.722, *p* = 0.472
Female: *n* (%)	61 (64)	34 (65)	**X^2^ = 0.020, *p* = 0.887
Disease duration (years): mean (SD)	18.2 (9.6)	/	/
Previous ON: *n* (%)	31 (33)	/	/
Disease course: RRMS: *n* (%) PMS: *n* (%)	78 (82) 17 (18)	/	/
EDSS: median (min–max)	3.0 (0–8.0)	/	/
On DMTs: *n* (%) Platform: *n* Oral: Monoclonal antibodies: *n*	69 (73) 8 48 13	/	/

^*^Group comparison performed with Welch’s *t*-test; **group comparison performed with Pearson’s chi-squared test

Abbreviations: *PwMS* patients with multiple sclerosis; *HCs* healthy controls; *SD* standard deviation; *ON* optic neuritis; *RRMS* relapsing–remitting multiple sclerosis; *PMS* progressive multiple sclerosis; *EDSS* Expanded Disability Status Scale; *DMTs* disease-modifying therapies; Platform DMTs included interferon-beta, and glatiramer-acetate; Oral DMTs included teriflunomide, dimethyl fumarate, and fingolimod; monoclonal antibodies DMTs included natalizumab, rituximab, ocrelizumab, and alemtuzumab

### Group comparisons

PwMS and HCs did not differ in age (*p* = 0.47), and sex distribution (*p* = 0.89).

Compared to HCs, PwMS showed lower pRNFL thickness (*p* < 0.0001), and lower GCIPL volume (*p* < 0.0001); there were no significant differences in INL volume (*p* = 0.06) (Table 2).

**Table 2:** OCT and MRI cohort’s characteristics

OCT measures	PwMS (*n* = 95)	HCs (*n* = 52)	Between-group comparison
pRNFL (μm): mean (SD)	92.9 (14.0)	100.9 (7.4)	*t = – 4.523, ***p* < 0.0001**
GCIPL (mm^3^): mean (SD)	1.87 (0.24)	2.04 (0.11)	*t = – 5.626, ***p* < 0.0001**
INL (mm^3^): mean (SD)	0.99 (0.06)	0.97 (0.06)	*t = 1.882, *p* = 0.063
MRI measures			
T2LV (ml): median (IQR)	6.6 (12.5)	0.1 (0.5)	**W = 4476, ***p* < 0.0001**
Total brain volume (ml): mean (SD)	1,013.4 (112.2)	1,128.5 (120.6)	***t = – 8.343, ***p* < 0.0001**
Total GM volume (ml): mean (SD)	565.2 (54.3)	629.3 (68.2)	***t = – 7.744, ***p* < 0.0001**
Total WM volume (ml): mean (SD)	449.6 (68.8)	499.5 (65.6)	***t = – 4.342, ***p* < 0.0001**
Cerebral cortex volume (ml): mean (SD)	418.6 (44.4)	463.8 (55.9)	***t = – 6.232, ***p* < 0.0001**
Deep GM volume (ml): mean (SD)	50.1 (6.0)	57.1 (6.2)	***t = – 7.925, ***p* < 0.0001**
Thalamic volume (ml): mean (SD)	12.4 (2.2)	14.2 (2.1)	***t = – 5.144, ***p* < 0.0001**
Caudate volume (ml): mean (SD)	6.3 (1.0)	6.9 (1.2)	***t = – 1.954, *p* = 0.0526
Putamen volume (ml): mean (SD)	8.4 (1.9)	9.7 (1.4)	***t = – 5.132, ***p* < 0.0001**
Hippocampal volume (ml): mean (SD)	7.2 (0.8)	8.3 (0.8)	***t = – 8.548, ***p* < 0.0001**
Cerebellar volume (ml): mean (SD)	119.6 (11.9)	138.0 (18.0)	***t = – 6.608, ***p* < 0.0001**

^*^Group comparison performed with Welch’s *t*-test; **group comparison performed with Mann–Whitney *U* test; ***group comparison performed with general linear model adjusting for age, sex, and total intracranial volume. Significant *p*-values are reported in bold

Abbreviations: *PwMS* patients with multiple sclerosis; *HCs* healthy controls; *pRNFL* peripapillary retinal nerve fiber layer; *GCIPL* ganglion cell-inner plexiform layer; *INL* inner nuclear layer; T2LV, T2-lesion volume; *IQR* interquartile range; *SD* standard deviation; *GM* gray matter; *WM* white matter

PwMS showed lower TBV than HCs, as well as lower total GM and WM volumes (all *p* < 0.0001) (Table 2).

### Associations between OCT and brain volumetric measurements

In PwMS, both pRNFL and GCIPL were positively associated with TBV (*β* = 0.170; *p* = 0.002 and *β* = 0.128; *p* = 0.04, respectively), and total GM volume (*β* = 0.252; *p* = 0.001 and *β* = 0.271; *p* = 0.002, respectively), but not with total WM volume.

When looking at different GM regions, both pRNFL and GCIPL were associated with the volumes of the cerebral cortex (*β* = 0.234; *p* = 0.003 and *β* = 0.273; *p* = 0.002, respectively), and thalamus (*β* = 0.214; *p* = 0.002 and *β* = 0.150; *p* = 0.04, respectively), while only GCIPL was associated with hippocampal volume (*β* = 0.235; *p* = 0.04) (Table 3).

**Table 3:** Association between OCT measures and brain volumes in PwMS

	pRNFL (*n* = 95)		GCIPL (*n* = 89)		INL (*n* = 89)	
	*β*	FDR-p	*β*	FDR-p	*β*	FDR-p
Total brain volume	0.170	**0.0020**	0.128	**0.0386**	– 0.079	0.5585
Total GM	0.252	**0.0013**	0.271	**0.0019**	0.041	0.6956
Total WM	0.079	0.1324	– 0.006	0.9114	– 0.160	**0.0130**
Cerebral cortex	0.234	**0.0031**	0.273	**0.0021**	0.080	0.5774
Thalamus	0.214	**0.0020**	0.150	**0.0408**	– 0.057	0.6000
Caudate	0.189	0.0876	0.175	0.08813	0.049	0.6956
Putamen	0.129	0.0876	0.075	0.3594	– 0.075	0.5774
Hippocampus	0.175	0.0876	0.235	**0.0408**	– 0.004	0.9657
Cerebellum	0.159	0.1176	0.187	0.0881	– 0.129	0.5643

Associations between brain volumes (dependent variable) and OCT measures (independent variable) were explored with general linear models, adjusting for age, sex, T2-lesion volume, and total intracranial volume. Significant *p*-values are reported in bold

Abbreviations: *pRNFL* peripapillary retinal nerve fiber layer; *GCIPL* ganglion cell-inner plexiform layer; *INL* inner nuclear layer; *β* standardized regression coefficient; *FDR* false discovery rate; *GM* gray matter; *WM* white matter

**Fig. 1:**
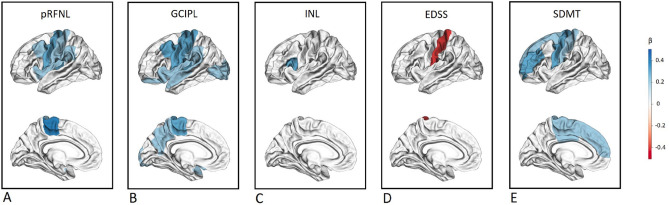
Associations of regional cortical volumes with OCT measures and with measures of disability. The effect size, expressed as standardized beta (*β*), is graphically displayed for each of the Desikan-Killiany atlas regions presenting significant association after multiple comparisons correction. Abbreviations: *pRNFL* peripapillary retinal nerve fiber layer; *GCIPL* ganglion cell-inner plexiform layer; *INL* inner nuclear layer; EDSS, Expanded Disability Status Scale; *SDMT* Symbol Digit Modalities Test; *β* standardized regression coefficient

When considering regional *cortical* volumes, mean pRNFL thickness showed extensive associations involving the frontal (*β* = 0.277; *p* = 0.007), occipital (*β* = 0.239; *p* = 0.01), insular (*β* = 0.227; *p* = 0.01), and parietal (*β* = 0.205; *p* = 0.01) lobes. Similarly, GCIPL volume was positively associated with cortical volume in the frontal (*β* = 0.295; p = 0.004), parietal (*β* = 0.251; *p* = 0.004), occipital (*β* = 0.244; *p* = 0.02), insular (*β* = 0.219; *p* = 0.002), and temporal (*β* = 0.194; *p* = 0.02) lobes (Fig. 1).

In contrast to pRNFL and GCIPL, INL volume was not associated with any measure of GM volume. Conversely, INL showed an inverse association with total WM volume (*β* = – 0.160; *p* = 0.01) (Table 3). In the cortical regional analysis, INL was not associated with the volume of any brain lobe, and only a regional association to the pars opercularis of the inferior frontal gyrus emerged (*β* = 0.305; *p* = 0.003) (Fig. 1).

Associations between OCT and MRI measures were not significantly altered after including in the models DMTs class as a covariate (Online Resource 2).

In the subgroup of patients with RRMS, correlations of pRNFL with TBV, total GM volume, cerebral cortex volume, and thalamic volume were also observed (Online Resource 3). No associations between OCT and MRI measures survived multiple comparisons correction in the subgroup of patients with progressive MS (*n* = 17).

Significant associations between OCT and MRI measures were confirmed in PwMS also when including disease duration and EDSS score as covariates in the multivariable regression models (Online Resources 5–6).

No association between OCT measures and brain volumes were found in HCs.

### Associations between OCT and WM lesions

In PwMS, both pRNFL and GCIPL were associated with total T2LV (*β* = – 0.327; *p* = 0.004 and *β* = – 0.397; *p* < 0.0001, respectively), as well as with T2LV in vast majority of brain lobes. The strongest association was measured with T2LV in the ORs (pRNFL: *β* = – 0.387; *p* = 0.001; GCIPL: *β* = – 0.471; *p* < 0.0001); the association remained significant after adjusting for total T2LV. INL volume was not associated with total, nor with lobar T2LV (Table 4).

**Table 4:** Association between T2-lesion load and OCT measures in PwMS

	pRNFL (*n* = 95)		GCIPL (*n*=89)		INL (*n*=89)	
	*β*	FDR-p	*β*	FDR-p	*β*	FDR-p
*Total T2LV	– 0.327	**0.0036**	– 0.397	** < 0.0001**	– 0.100	0.9759
Regional lesion volume						
*Temporal T2LV	– 0.253	**0.0198**	– 0.344	**0.0020**	– 0.045	0.9759
*Frontal T2LV	– 0.223	**0.0338**	– 0.301	**0.0062**	– 0.001	0.9950
*Parietal T2LV	– 0.268	**0.0157**	– 0.340	**0.0020**	– 0.010	0.9950
*Occipital T2LV	– 0.335	**0.0036**	– 0.426	**0.0001**	– 0.033	0.9759
*Cingulate T2LV	– 0.299	**0.0074**	– 0.272	**0.0129**	0.067	0.9759
*Insular T2LV	– 0.162	0.1160	– 0.222	**0.0367**	– 0.045	0.9759
*ORs T2LV	– 0.387	0.0010	– 0.471	< 0.0001	– 0.078	0.9759
**ORs T2LV	– 0.438	**0.0334**	– 0.464	**0.0186**	0.034	0.9759

*Association explored with univariable linear regression; **association explored with linear regression adjusting for total T2LV. Significant p-values are reported in bold

Abbreviations: *pRNFL* peripapillary retinal nerve fiber layer; *GCIPL* ganglion cell-inner plexiform layer; *INL* inner nuclear layer; *β* standardized regression coefficient; *FDR* false discovery rate; *T2LV* T2-weighted hyperintense lesion volume; *ORs* optic radiations

No association between OCT measures and global/regional T2LV was present in HCs.

Overall, lesion volume in the ORs (taken as marker of retrograde degeneration *in the visual pathway*) and GM fraction (taken as marker of *global* neurodegeneration in the brain) showed a similar strength of associations with both pRNFL and GCIPL (Table 5).

**Table 5:** Association of ORs lesions and GM fraction with the OCT measures of neuroaxonal loss

	pRNFL		GCIPL	
Independent variables	β	*p*-value	*β*	*p*-value
T2LV in ORs	– 0.393	**0.0004**	– 0.383	**0.0006**
GM fraction	0.337	**0.0145**	0.380	**0.0075**
Adjusted *R*^2^	0.2170		0.2519	

The OCT measures were each time dependent variables, and the lesion volume in the ORs as well as the GM fraction were independent variables in the linear regression models, adjusting for age, sex, total T2LV, and total intracranial volume. Significant *p*-values are reported in bold

Abbreviations: *pRNFL* peripapillary retinal nerve fiber layer; *GCIPL* ganglion cell-inner plexiform layer; *β* standardized regression coefficient; *T2LV* T2-weighted hyperintense lesion volume; *ORs* optic radiations; *GM* gray matter

### Associations among OCT, MRI and clinical measures

Both pRNFL and GCIPL were inversely associated with the EDSS (*β* = – 0.422; *p* < 0.0001 and *β* = – 0.519; *p* =  < 0.0001, respectively), and positively associated with the SDMT (*β* = 0.377; *p* = 0.0002 and *β* = 0.402; *p* < 0.0001, respectively), while INL did not significantly correlate with clinical measures.

**Fig. 2:**
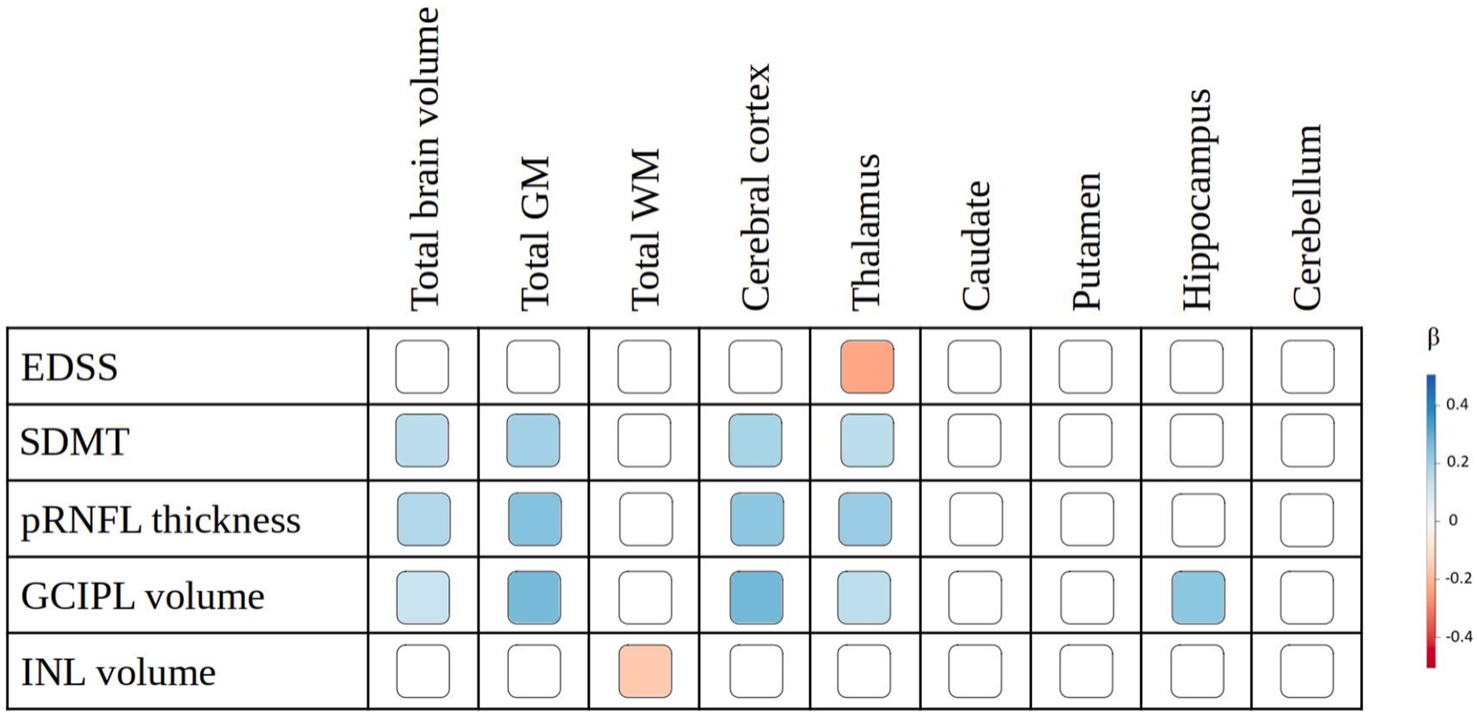
Associations of MRI measures with OCT measures and with measures of disability. The effect size, expressed as standardized beta (*β*), is graphically displayed for each brain volume presenting significant association after multiple comparisons correction. Abbreviations: EDSS, Expanded Disability Status Scale; *SDMT* Symbol Digit Modalities Test; *pRNFL* peripapillary retinal nerve fiber layer; *GCIPL* ganglion cell-inner plexiform layer; *INL* inner nuclear layer; *β* standardized regression coefficient; *GM* gray matter; *WM* white matter

EDSS and SDMT were associated with thalamic volume (*β* = – 0.233; *p* = 0.01 and *β* = 0.153; *p* = 0.04, respectively), while only SDMT showed association with TBV (*β* = 0.152; *p* = 0.02), total GM volume (*β* = 0.198; *p* = 0.02), and cortical volume (*β* = 0.188; *p* = 0.04). In regional cortical analysis, the EDSS showed an association limited to the volume of post-central gyrus (*β* = – 0.308; *p* = 0.04), while the SDMT presented more extensive associations, involving areas in the frontal, parietal, and cingulate cortex. The overlap between the brain structures showing associations with EDSS/SDMT and those associated with OCT measures is graphically displayed in Figs. 1 and 2.

## Discussion

In this study, we found that the OCT measures of neuroaxonal loss (pRNFL, GCIPL) in PwMS reflect GM rather than WM integrity. Both pRNFL and GCIPL showed also associations with the burden of focal lesion damage, especially in the ORs (in line with retrograde degeneration in the visual pathway). Overall, the strength of the OCT-associations was similar for GM fraction and for lesion volume in the ORs, suggesting that global CNS neurodegeneration and retrograde degeneration along the visual pathway may contribute similarly to pRNFL and GCIPL thinning in MS.

Notably, pRNFL and GCIPL were associated with volumes of brain structures such as the thalamus and the cerebral cortex, that are related to physical and cognitive disability in MS.

In contrast to the OCT measures of neuroaxonal loss, INL showed no associations with GM integrity nor with WM lesions, but an inverse association with WM volume.

Our results are in line with previous studies showing associations between retinal measures of neuroaxonal loss and GM volume [6, 8, 11].⁠ The association of pRNFL and GCIPL with GM integrity provides information on key pathological processes of MS. Indeed, GM is significantly affected in PwMS, even from the earliest disease stages, with rates of decline greater than WM [24, 25] Furthermore, GM atrophy is recognized as a pivotal process underlying disease progression in MS, reflecting disability to a greater extent than WM loss [26].⁠ Considering that MRI monitoring of GM integrity at the individual patient level is still very challenging in clinical practice, [26, 27] OCT may represent a valuable marker of GM damage and neurodegeneration in MS.

The relationship between OCT measures of neuroaxonal loss and thalamic volume is also of interest, since thalamic atrophy has been proposed as a marker of the net accumulation of MS-related damage throughout the entire CNS [28].

In our cohort, pRNFL and GCIPL correlated also with the volume of the entire cerebral cortex, as well as regionally with the integrity of several cortical areas. Cortical atrophy is common in MS, and it is considered to progress largely in a non-random manner, correlating with both physical and cognitive disability [29, 30].⁠ Interestingly, we found that the areas showing association with OCT measures were not limited to the visual cortex, but rather involved diffuse regions in the frontal, parietal, temporal, and insular lobes, in a pattern more widespread than previously reported [12–14].⁠

To further investigate whether the brain volumes associated with OCT measures were clinically-relevant, we also explored the association between brain volumes and measures of physical and cognitive disability. Remarkably, the brain volumes associated with measures of both physical and cognitive disability significantly overlapped with those correlating with OCT measures of neuroaxonal loss. Specifically, after adjusting for age, sex, TIV, and lesion load, physical disability was associated with thalamic volume, while cognitive disability was associated with TBV, and the volumes of total GM, thalamus and cerebral cortex; all of these brain compartments were also associated with both pRNFL and GCIPL.

In line with previous observations [5, 6, 11],⁠ OCT measures of neuroaxonal loss correlated also with WM lesion load, most notably in the ORs. Interestingly, in contrast with a recent study which reported an association with T2LV only for pRNFL thickness, in our cohort GCIPL volume was also associated with T2LV (especially in the ORs), suggesting that retrograde degeneration reaches the GCIPL, as previously proposed [31]⁠.

Overall, we found very similar associations of the OCT measures with both WM lesion burden in the ORs and GM atrophy, suggesting a similar contribution of both mechanisms to the retinal changes. However, these variables, together with age, sex, and TIV explained only 20–25% of the variability in OCT measures in our models. Thus, other factors, such as biological variability, subclinical demyelination of the optic nerves as well as subclinical inflammatory and neurodegenerative processes in the retina (independently of the brain), may be important contributors to the observed OCT changes. To exclude that the observed OCT-MRI associations were critically driven by definite subsets of PwMS, we conducted sensitivity analyses to explore the effect of disease phenotype, disease duration, and EDSS score on such associations. Overall, substantial OCT-MRI correlations were confirmed in patients with RRMS, as well as in the entire cohort of PwMS when adjusting for the effect of disease duration and disability severity. Notably, while no OCT-MRI associations survived multiple comparisons correction in patients with progressive MS, such results are likely significantly influenced by the small sample size in this subgroup.

In our cohort, INL volume showed a different behavior than pRNFL and GCIPL, exhibiting no associations with GM integrity and inverse correlation with WM volume. This result, which is in line with a previous study, [32]⁠ may support the role of INL thickening as an indicator of inflammatory processes in the WM—ultimately resulting in WM atrophy as previously proposed [4, 32].⁠

Overall, we found that both pRNFL and GCIPL exhibited very similar patterns of associations not only with the MRI measures, but also with clinical disability. This is partially in contrast with a previous study suggesting that GCIPL may be a more reliable measure of neurodegeneration than pRNFL [11].⁠ Notably, these results may be of practical interest, since the assessment of pRNFL can be more easily implemented in clinical practice, and may present less inter-center variability than GCIPL [33]⁠.

No associations between OCT and MRI measures emerged in HCs, supporting the independence of the results found in PwMS from physiological, age-related mechanisms of retinal and brain neurodegeneration. Moreover, the lack of association between the incidental WM hyperintensities detected in HCs and retinal integrity may suggest that those lesions are less likely to produce retrograde degeneration, although it should be noted that the total lesion load was much lower in HCs than in PwMS, and a potential critical threshold may not have been reached.

Our study has limitations, the most relevant being its cross-sectional design, which does not allow the investigation of the temporal dynamics of the relationship between retinal and brain atrophy. Moreover, we did not include measures of spinal cord damage. Additionally, exploring a high number of associations between OCT and MRI measures may per se increase the chances for significant results; however, to address this point all results were corrected for multiple comparisons.

Strengths of our study are the comprehensive assessment of changes in the retina and brain, with measures reflecting both neurodegeneration and inflammation at a global but also regional level, as well as the combination of last generation OCT and high-resolution MRI, both obtained with standardized protocols. Despite the study being conducted in a single center, the PwMS included were participants of a representative MS-cohort, characterized by a variety of ages, degrees of disability, and treatments. Thus, taking into account the above mentioned limitations, the results of this study may be generalizable to a larger population with MS.

To conclude, we showed that OCT measures of retinal neuroaxonal loss (pRNFL and GCIPL) reflect the accumulation of GM loss in PwMS, independently of lesion load in the whole brain and in the ORs. The overlap between the brain regions associated with retinal atrophy and those associated with physical and cognitive disability underlines the clinical relevance of OCT measures, and highlights the role of pRNFL and GCIPL as markers of neurodegeneration and disability in MS.

## Supplementary Information

Below is the link to the electronic supplementary material.

Supplementary file1 (DOCX 23 KB)

## Data Availability

Data are available from the corresponding author upon reasonable request.
