# Supplementary material for: Optical coherence tomography reflects clinically relevant gray matter damage in patients with multiple sclerosis
Source: J Neurol. 2023 Jan 10;270(4):2139–48. doi: 10.1007/s00415-022-11535-8 (PMC10025239; doi:10.1007/s00415-022-11535-8)
Supplement: Supplementary file 1 — Supplementary file1 (DOCX 23 KB) [file 415_2022_11535_MOESM1_ESM.docx]

**Online Resources**

| **Subject_ID** | **Left eye** | **Right eye** |
| --- | --- | --- |
| MS_#2650 | Technical issue | Technical issue |
| MS_#2674 | Incidental finding | Optic neuritis |
| MS_#2787 | Technical issue | Optic neuritis |
| MS_#2873 | Technical issue | Incidental finding |
| MS_#2885 | Optic neuritis | Technical issue |
| MS_#5440 | Optic neuritis | Incidental finding |
| HC_#6648 | Incidental finding | Technical issue |
| HC_#6666 | Incidental finding | Technical issue |

**Online Resource 1. List of subjects for which GCIPL and INL measures were excluded, and reasons for exclusion.**

|  | **GCIPL (n=89)** | | | **RNFL (n=95)** | | | **INL (n=89)** | | | **EDSS (n=95)** | | | **SDMT (n=95)** | | |
| --- | --- | --- | --- | --- | --- | --- | --- | --- | --- | --- | --- | --- | --- | --- | --- |
|  | β | p-value | p-adjusted (FDR) | β | p-value | p-adjusted (FDR) | β | p-value | p-adjusted (FDR) | β | p-value | p-adjusted (FDR) | β | p-value | p-adjusted (FDR) |
| Total brain volume | 0.126 | 0.0197 | **0.0461** | 0.159 | 0.0022 | **0.0066** | -0.086 | 0.0905 | 0.4073 | -0.116 | 0.0593 | 0.2669 | 0.174 | 0.0010 | **0.0077** |
| Total GM | 0.265 | 0.0003 | **0.0027** | 0.234 | 0.0009 | **0.0041** | 0.047 | 0.5085 | 0.6538 | -0.104 | 0.2191 | 0.4146 | 0.226 | 0.0017 | **0.0077** |
| Total WM | -0.018 | 0.7494 | 0.7494 | 0.067 | 0.2176 | 0.2176 | -0.178 | 0.0004 | **0.0040** | -0.093 | 0.1427 | 0.4146 | 0.093 | 0.0923 | 0.1385 |
| Cerebral cortex | 0.263 | 0.0008 | **0.0036** | 0.216 | 0.0041 | **0.0093** | 0.080 | 0.2907 | 0.5529 | -0.058 | 0.5185 | 0.5589 | 0.205 | 0.0077 | **0.0231** |
| Thalamus | 0.156 | 0.0205 | **0.0461** | 0.221 | 0.0005 | **0.0040** | -0.062 | 0.3326 | 0.5529 | -0.232 | 0.0020 | **0.0180** | 0.157 | 0.0185 | **0.0356** |
| Caudate | 0.177 | 0.0662 | 0.0851 | 0.196 | 0.0568 | 0.1022 | 0.045 | 0.6152 | 0.6921 | -0.100 | 0.4068 | 0.5230 | 0.243 | 0.0198 | **0.0356** |
| Putamen | 0.074 | 0.3289 | 0.3700 | 0.106 | 0.1365 | 0.1754 | -0.063 | 0.3686 | 0.5529 | -0.049 | 0.5589 | 0.5589 | 0.092 | 0.2024 | 0.2602 |
| Hippocampus | 0.223 | 0.0325 | 0.0585 | 0.155 | 0.1176 | 0.1754 | -0.028 | 0.7759 | 0.7759 | -0.138 | 0.2304 | 0.4146 | 0.081 | 0.4210 | 0.4210 |
| Cerebellum | 0.198 | 0.0483 | 0.0725 | 0.132 | 0.1865 | 0.2098 | -0.094 | 0.3206 | 0.5529 | -0.115 | 0.3228 | 0.4842 | 0.121 | 0.2355 | 0.2649 |

***Online Resource 2. Associations of OCT measures and clinical measures with brain volumes, adjusting for age, sex, total intracranial volume (TIV), total lesion volume, and treatment class.*** *Treatment class were the following: platform DMTs (including: interferon-beta, and glatiramer-acetate); oral DMTs (including: teriflunomide, dimethyl fumarate, and fingolimod); monoclonal antibodies (including: natalizumab, rituximab, ocrelizumab, and alemtuzumab); untreated patients.*

|  | **pRNFL (n=78)** | | | **GCIPL (n=74)** | | | **INL (n=74)** | | |
| --- | --- | --- | --- | --- | --- | --- | --- | --- | --- |
|  | β | p-value | p-adjusted (FDR) | β | p-value | p-adjusted (FDR) | β | p-value | p-adjusted (FDR) |
| Total brain volume | 0.136 | 0.0206 | **0.0491** | 0.056 | 0.3420 | 0.4506 | -0.107 | 0.0558 | 0.1674 |
| Total GM | 0.216 | 0.0068 | **0.0306** | 0.192 | 0.0192 | 0.0795 | 0.033 | 0.6778 | 0.6778 |
| Total WM | 0.044 | 0.4665 | 0.4665 | -0.066 | 0.2849 | 0.4506 | -0.199 | 0.0005 | **0.0045** |
| Cerebral cortex | 0.196 | 0.0218 | **0.0491** | 0.195 | 0.0259 | 0.0795 | 0.088 | 0.0302 | 0.1359 |
| Thalamus | 0.200 | 0.0044 | **0.0306** | 0.088 | 0.2256 | 0.4506 | -0.144 | 0.1007 | 0.1980 |
| Caudate | 0.188 | 0.0785 | 0.1226 | 0.231 | 0.0265 | 0.0795 | -0.070 | 0.4916 | 0.6019 |
| Putamen | 0.113 | 0.1703 | 0.2190 | 0.055 | 0.5315 | 0.5315 | -0.084 | 0.3160 | 0.4740 |
| Hippocampus | 0.095 | 0.4104 | 0.4617 | 0.102 | 0.4005 | 0.4506 | -0.073 | 0.5350 | 0.6019 |
| Cerebellum | 0.191 | 0.0817 | 0.1226 | 0.095 | 0.3838 | 0.4506 | -0.166 | 0.1100 | 0.1980 |

***Online Resource 3. Associations of OCT measures with brain volumes in patients with relapsing-remitting multiple sclerosis, adjusting for age, sex, total intracranial volume (TIV), and total lesion volume.***

|  | **pRNFL (n=17)** | | | **GCIPL (n=15)** | | | **INL (n=15)** | | |
| --- | --- | --- | --- | --- | --- | --- | --- | --- | --- |
|  | β | p-value | p-adjusted (FDR) | β | p-value | p-adjusted (FDR) | β | p-value | p-adjusted (FDR) |
| Total brain volume | 0.231 | 0.0645 | 0.1935 | 0.277 | 0.0698 | 0.2450 | 0.001 | 0.9963 | 0.9963 |
| Total GM | 0.434 | 0.0456 | 0.1935 | 0.444 | 0.0929 | 0.2450 | -0.025 | 0.9215 | 0.9963 |
| Total WM | 0.128 | 0.2012 | 0.3870 | 0.180 | 0.1361 | 0.2450 | -0.012 | 0.9120 | 0.9963 |
| Cerebral cortex | 0.457 | 0.0323 | 0.1935 | 0.481 | 0.0632 | 0.2450 | -0.168 | 0.4940 | 0.9279 |
| Thalamus | 0.179 | 0.3451 | 0.5177 | 0.290 | 0.2000 | 0.3000 | 0.200 | 0.3203 | 0.9279 |
| Caudate | 0.114 | 0.6892 | 0.7754 | 0.169 | 0.5960 | 0.6700 | 0.484 | 0.0558 | 0.5022 |
| Putamen | 0.094 | 0.4642 | 0.5968 | 0.057 | 0.6544 | 0.6700 | 0.072 | 0.5155 | 0.9279 |
| Hippocampus | 0.361 | 0.2150 | 0.3870 | 0.467 | 0.131 | 0.2450 | -0.032 | 0.9110 | 0.9963 |
| Cerebellum | 0.0361 | 0.8950 | 0.8950 | 0.144 | 0.670 | 0.6700 | 0.1980 | 0.5000 | 0.9279 |

***Online Resource 4. Associations of OCT measures with brain volumes in patients with progressive multiple sclerosis, adjusting for age, sex, total intracranial volume (TIV), and total lesion volume.***

|  | **pRNFL (n=95)** | | | **GCIPL (n=89)** | | | **INL (n=89)** | | |
| --- | --- | --- | --- | --- | --- | --- | --- | --- | --- |
|  | β | p-value | p-adjusted (FDR) | β | p-value | p-adjusted (FDR) | β | p-value | p-adjusted (FDR) |
| Total brain volume | 0.155 | 0.0025 | **0.0068** | 0.106 | 0.0463 | 0.1042 | -0.063 | 0.2049 | 0.6147 |
| Total GM | 0.250 | 0.0004 | **0.0036** | 0.180 | 0.0072 | **0.0324** | 0.066 | 0.3521 | 0.6740 |
| Total WM | 0.051 | 0.3397 | 0.3397 | -0.049 | 0.3764 | 0.4235 | -0.158 | 0.0015 | **0.0135** |
| Cerebral cortex | 0.238 | 0.0018 | **0.0068** | 0.279 | 0.0005 | **0.0045** | 0.099 | 0.1936 | 0.6147 |
| Thalamus | 0.183 | 0.0030 | **0.0068** | 0.104 | 0.1076 | 0.1614 | -0.031 | 0.6076 | 0.7261 |
| Caudate | 0.216 | 0.0380 | 0.0684 | 0.178 | 0.0756 | 0.1361 | 0.043 | 0.6454 | 0.7261 |
| Putamen | 0.120 | 0.0986 | 0.1268 | 0.060 | 0.4419 | 0.4419 | -0.054 | 0.4493 | 0.6740 |
| Hippocampus | 0.180 | 0.0702 | 0.1053 | 0.244 | 0.0214 | 0.0642 | -0.007 | 0.9467 | 0.9467 |
| Cerebellum | 0.121 | 0.2197 | 0.2472 | 0.150 | 0.1415 | 0.1819 | -0.078 | 0.4088 | 0.6740 |

***Online Resource 5. Associations of OCT measures and clinical measures with brain volumes, adjusting for age, sex, total intracranial volume (TIV), total lesion volume, and disease duration.***

|  | **pRNFL (n=95)** | | | **GCIPL (n=89)** | | | **INL (n=89)** | | |
| --- | --- | --- | --- | --- | --- | --- | --- | --- | --- |
|  | β | p-value | p-adjusted (FDR) | β | p-value | p-adjusted (FDR) | β | p-value | p-adjusted (FDR) |
| Total brain volume | 0.159 | 0.0032 | **0.0096** | 0.094 | 0.1073 | 0.1931 | -0.065 | 0.1880 | 0.5640 |
| Total GM | 0.255 | 0.0006 | **0.0054** | 0.270 | 0.0008 | **0.0036** | 0.071 | 0.3133 | 0.6726 |
| Total WM | 0.055 | 0.3306 | 0.3306 | -0.070 | 0.2473 | 0.3710 | -0.165 | 0.0010 | **0.0090** |
| Cerebral cortex | 0.248 | 0.0018 | **0.0081** | 0.296 | 0.0006 | **0.0036** | 0.103 | 0.1695 | 0.5640 |
| Thalamus | 0.162 | 0.0109 | **0.0245** | 0.050 | 0.4695 | 0.5282 | -0.031 | 0.5940 | 0.7613 |
| Caudate | 0.180 | 0.0974 | 0.1461 | 0.211 | 0.0512 | 0.1247 | 0.038 | 0.6767 | 0.7613 |
| Putamen | 0.142 | 0.0601 | 0.1082 | 0.045 | 0.5939 | 0.5939 | -0.054 | 0.4484 | 0.6726 |
| Hippocampus | 0.161 | 0.1190 | 0.1494 | 0.219 | 0.0554 | 0.1247 | 0.012 | 0.9019 | 0.9019 |
| Cerebellum | 0.158 | 0.1328 | 0.1494 | 0.093 | 0.3920 | 0.5040 | -0.072 | 0.4351 | 0.6726 |

***Online Resource 6. Associations of OCT measures and clinical measures with brain volumes, adjusting for age, sex, total intracranial volume (TIV), total lesion volume, and EDSS.***
